# Management of Plastic Bronchitis Using α-Chymotrypsin: A Novel Treatment Modality

**DOI:** 10.7759/cureus.13551

**Published:** 2021-02-25

**Authors:** Lijuan Xiong, Xiaoping Rao, Xin Peng, Gaoping Zhang, Hong Liu

**Affiliations:** 1 Department of Emergency, The Affiliated Children's Hospital of Nanchang University, Nanchang, CHN; 2 Department of Pediatric Intensive Care Unit, The Affiliated Children's Hospital of Nanchang University, Nanchang, CHN; 3 Department of Otolaryngology, The Affiliated Children's Hospital of Nanchang University, Nanchang, CHN; 4 Department of Pneumology, The Affiliated Children's Hospital of Nanchang University, Nanchang, CHN

**Keywords:** plastic bronchitis, alpha-chymotrypsin, treatment, case report

## Abstract

Plastic bronchitis (PB) is a rare pediatric respiratory disease, characterized by the formation of obstructive casts in the bronchial tract that causes partial or extensive airway obstruction, leading to obstructive dyspnea mimicking a foreign body in the trachea. The clinical presentation and radiologic examination of plastic bronchitis are nonspecific, and the confirmation of the diagnosis is only possible via the direct observation of the casts via bronchoscopy or expectorating. So far, no effective treatment for PB has been demonstrated in controlled clinical trials, and presently, there are no reports regarding the use of α-chymotrypsin as a treatment modality. α-Chymotrypsin, as a mucolytic agent, liquefies the mucus and decreases the viscosity of sputum by acting directly on mucus. Here, we report a PB case that is associated with influenza A virus infection, developing in an eight-year-old boy. The diagnosis of PB was confirmed via cast observation following its removal via bronchoscopy. Specifically, the casts were successfully removed via bronchoscopy coupled with endotracheal instilled α-chymotrypsin. Thereafter, the patient gradually improved and successfully extubated. In the clinical follow-up, the patient was asymptomatic and without recurrent casts. Therefore, α-chymotrypsin may be one modality of treatment to remove casts in PB.

## Introduction

Plastic bronchitis (PB) is characterized by the formation of bronchitis casts, which causes partial or extensive airway obstruction. It occurs at any age, without a seasonal, regional, or epidemic trend. It is reported that PB has a high mortality rate according to the current literature, reaching approximately 28-60% in patients with congenital heart disease (CHD) and 6-7% in non-cardiac patients [1-3]. Recent studies have shown that several conditions, including CHD, Fontan operation, asthma, cystic fibrosis, acute chest pain syndrome in sickle cell disease, and influenza virus infection, are associated with PB [4-8]. Specifically, influenza is the most common infectious disease that is associated with PB, which is a serious and fatal pulmonary complication that is primarily seen in children [8]. To date, the influenza viruses that predominantly cause PB are influenza A and influenza B viruses, and it has been suggested that influenza A is more likely to cause serious PB complications than other common influenza viruses given that it can induce bronchial hyperresponsiveness and more serious damage to mucociliary clearance ability [9].

Considering the fact that PB is a severe and life-threatening disease owing to casts blocking the airway, to reduce mortality, it is crucial to remove the casts. In previous studies, several PB treatment methods, including chest physiotherapy and the use of mucolytic agents, fibrinolytic agents, and anti-inflammatory drugs, have been reported [4,6,10]. However, no effective treatment for PB has been demonstrated in controlled clinical trials, and there are no reports regarding the use of α-chymotrypsin as a treatment modality. α-Chymotrypsin has been widely used to accelerate the repair of traumatic injuries, burns, and to alleviate sciatica because of its anti-inflammatory, antioxidative, fibrinolytic, anti-edematous, and anti-infective properties [11]. Clinically, it is also used to liquefy the mucus and decrease the viscosity of sputum due to its direct mucolytic effect [12]. Recently, a PB patient admitted to our hospital had no significant clinical improvement after being given timely treatment including bronchoscopy, antibiotics, anti-inflammatory, nebulized bronchodilators, intensive chest physiotherapy, and intravenous mucosolvan. Since α-chymotrypsin has been used clinically to liquefy mucus, and the bronchial casts of the patient were mainly composed of mucus, we directly instilled α-chymotrypsin onto the casts and successfully remove the casts by continuous endotracheal suction. Therefore, in this case report, a patient with PB associated with influenza A virus infection is presented, and the experience using α-chymotrypsin coupled with bronchoscopy for cast removal from the lungs of this patient is described.

## Case presentation

**Figure 1:**
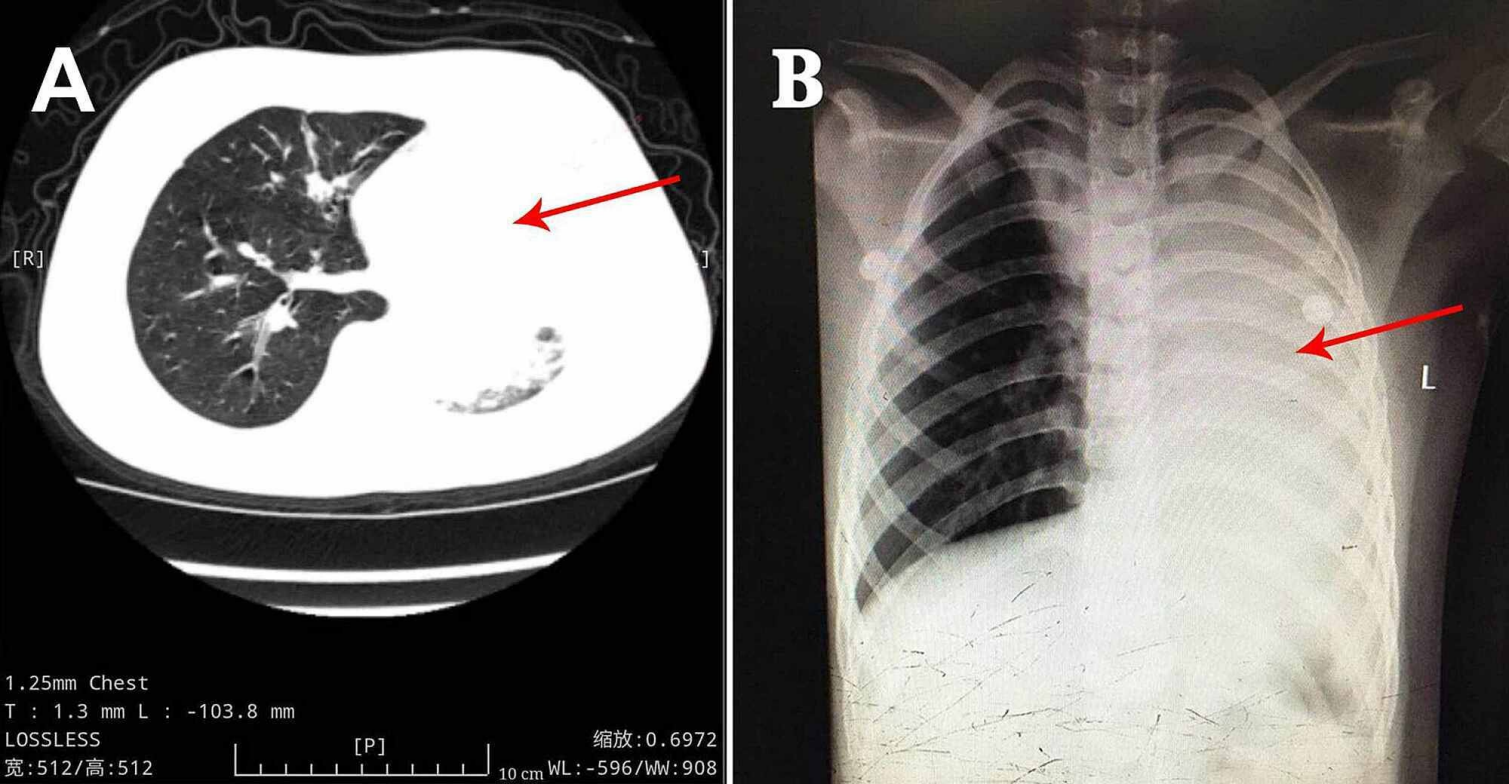
Primary Chest Computerized Tomography and Chest Radiograph (A) Chest CT at the local hospital showed atelectasis in the left lung and pneumonia in the right lung and (B) chest X-ray after mechanical ventilation revealed pneumonia in both lung lobes and atelectasis in the left lung.

**Figure 2:**
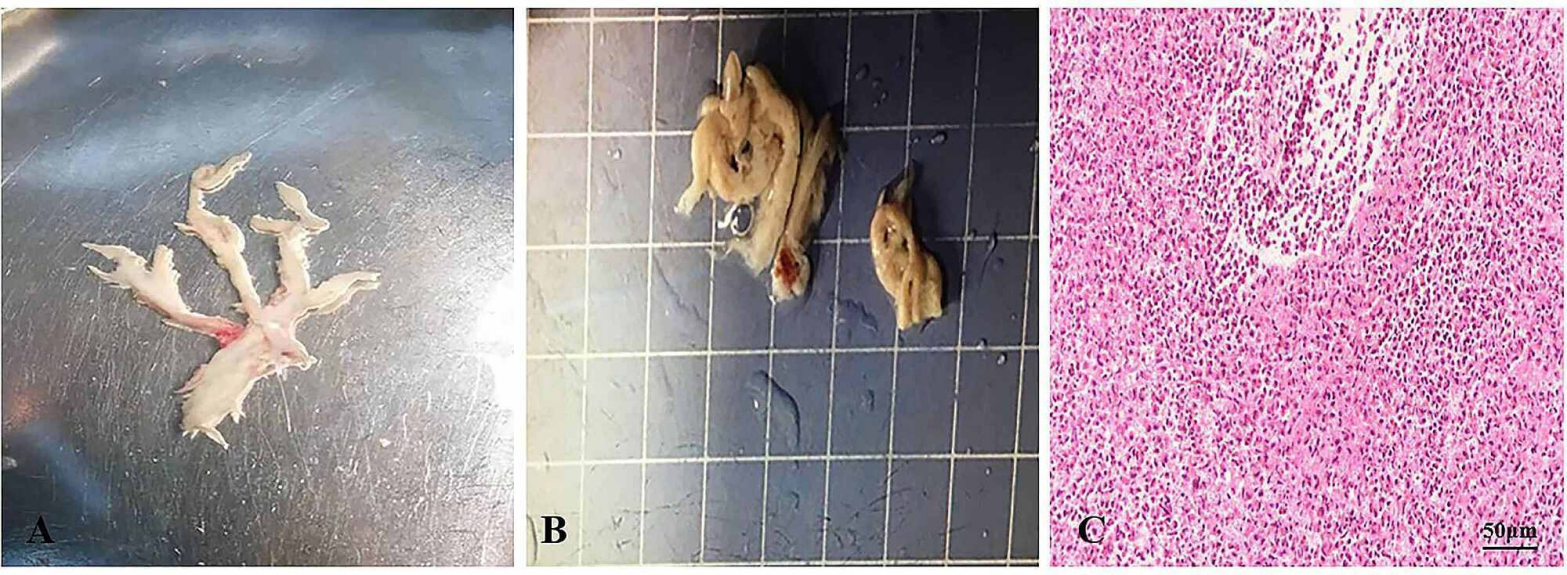
Findings of Bronchoscopy and Histopathological Examinations (A) Bronchoscopy showed complete obstruction of the left main bronchus by a bronchial tree-like cast; (B) casts were extracted using a sputum suction tube; (C) hematoxylin-eosin staining of casts shows inflammatory necrotic tissue, mucin, numerous neutrophils, and eosinophils (40× magnification).

A previously healthy eight-year-old boy, without the history of wheezing and foreign body inhalation, was transferred to the emergency center of Jiangxi Provincial Children's Hospital from a basic-level hospital with a two-day history of fever, cough, and progressive dyspnea. His body temperature was approximately 38 °C. He had been previously treated with antibiotics at a local hospital without improvement except the normalized body temperature. Subsequently, he underwent chest computed tomography (CT) scans, which revealed atelectasis in the left lung and a foreign body in the left upper bronchus, along with pneumonia in the right lung (Figure 1A). In our emergency department, physical examination showed clouding of consciousness, cyanosis, rales, and diminished breath sounds in both lungs. Other parameters were as follows: body temperature, 36.9 °C; heart rate, 150 beats/minute; respiratory rate, 46 breaths/minutes; percutaneous oxygen saturation, approximately 70% under high-flow oxygen therapy. Since the local chest CT scan and clinical manifestations indicated a high possibility of foreign body inhalation, urgent bronchoscopy was performed. This procedure did not reveal the presence of any foreign body in the trachea of this patient but showed the presence of white gelatinous secretions that completely blocked the left main bronchus. Upon removal, it was observed that the secretions are shaped like a bronchial tree (Figure 2A). However, after this cast removal followed by bronchial lavage, there was no significant improvement in oxygen saturation, which fluctuated between 70% and 80%. The patient was admitted to the pediatric intensive care unit and intubated immediately.

After the observation of the cast that was removed via bronchoscopy, PB diagnosis was confirmed. Further, histopathology indicated that the casts predominantly comprised of inflammatory necrotic tissue, mucin, numerous neutrophils, and eosinophils (Figure 2C). Chest X-ray revealed pneumonia in both lung lobes and atelectasis in the left lung (Figure 1B), suggesting airway blockage by residual casts. Furthermore, complete blood count revealed the following: white blood cells, 13.11×109 cells/L (leukocytosis); neutrophils, 85.4%; hemoglobin, 145 g/L; and platelets, 350×1012/L. The blood biochemistry results of this patient were as follows: C-reactive protein (CRP), 42.6 mg/L; IgE, 908.4 IU/ml; IgA, 1.04 IU/ml; IgM, 0.62 IU/ml; and IgG, 12.15 IU/ml. As PB diagnosis is confirmed, along with extensive inflammatory necrotic tissue in the casts, we started treatments as follows: imipenem-cilastatin sodium (30 mg/kg, q6h) for anti-infection; azithromycin (10 mg/kg, qd); intravenous methylprednisolone (1 mg/kg, q12h); nebulized dexamethasone (5 mg, q6h) for anti-inflammation; nebulized bronchodilators, intensive chest physiotherapy, and intravenous mucosolvan for mucolytic therapy. However, oxygen saturation still fluctuated at approximately 80% without improvement for the next two days. Sputum etiology examination was positive for influenza A virus and negative for other viruses and *Mycoplasma pneumoniae* RNA, as well as fungi and bacteria. Additionally, blood cultures for bacteria and fungi were also negative.

**Figure 3:**
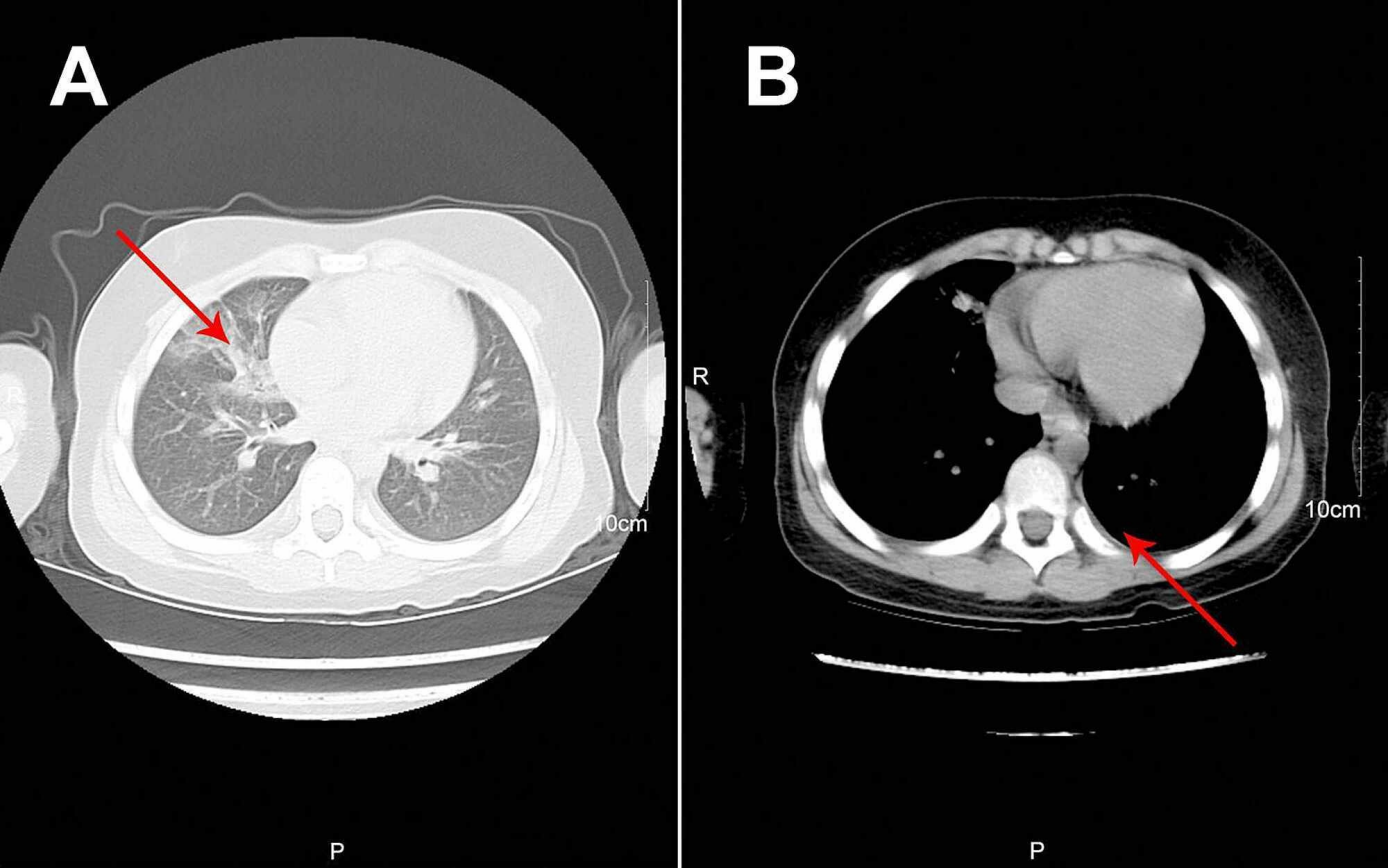
Reexamination of Chest Computerized Tomography (A) Chest CT reexamination after extubation showed full expansion of the left lung with pleural effusion on the left thorax and bilateral pneumonia and (B) chest CT for the first follow-up visit showed increased lung markings.

**Figure 4:**
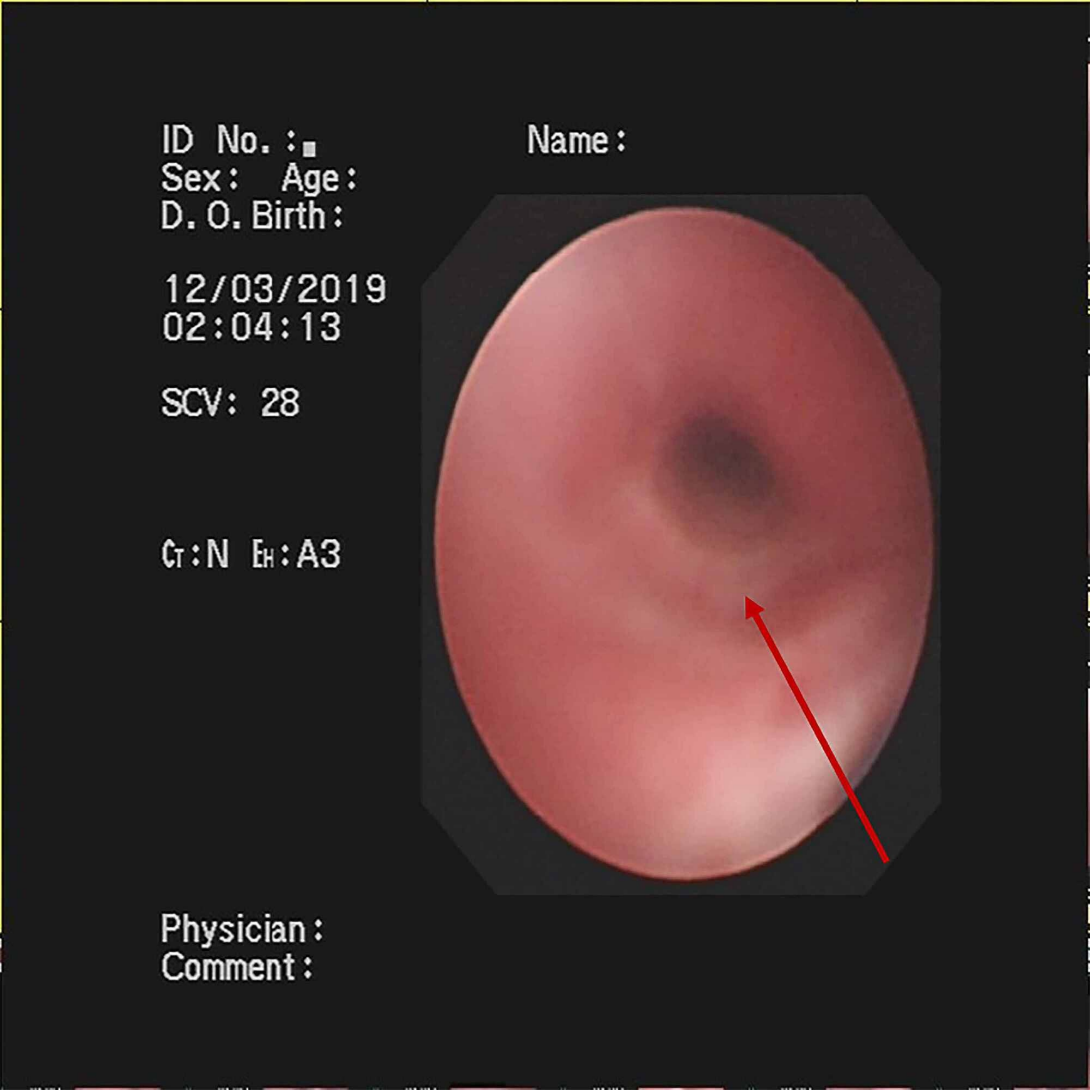
Findings of Fiberoptic Bronchoscopy Fiberoptic bronchoscopy revealed endobronchial intima infection without casts.

**Figure 5:**
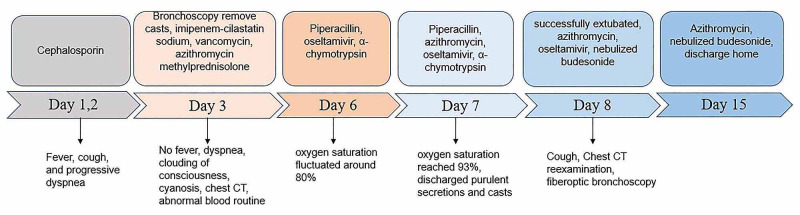
The Timeline of This Case

Treatment regimens were adjusted based on the etiological results. Imipenem-cilastatin sodium and vancomycin were discontinued; further, the patient was administered with piperacillin (50 mg/kg, bid), and added Oseltamivir for anti-influenza A virus. Azithromycin (10 mg/kg, qd), intravenous methylprednisolone (1 mg/kg, q12h), nebulized dexamethasone (5 mg, q6h), nebulized bronchodilators, intensive chest physiotherapy, and intravenous mucosolvan were continued. However, there was still no improvement. Considering the histopathology showed the casts predominantly comprised of mucin, and the mucolytic agent mucosolvan, as well as bronchodilators, were not effective, we used a mucolytic agent-α-chymotrypsin, which has been reported to liquefy the mucus. α-chymotrypsin (2000 IU, 5 mL saline) was intratracheally instilled twice a day via the endotracheal tube, supplemented by frequent intratracheal suction and chest physiotherapy. Consequently, abundant purulent secretions and casts were discharged (Figure 2B); further, the oxygen saturation increased to 93% and there were enhanced breath sounds in both lungs. The patient was successfully extubated with gradual improvement in his condition. Subsequently, the patient received oral azithromycin (10 mg/kg, three times per week) and nebulized budesonide (1 mg added in 2 mL saline, twice daily). Chest CT reexamination showed the full expansion of the left lung with pleural effusion on the left thorax and bilateral pneumonia (Figure 3A). Additionally, fiberoptic bronchoscopy revealed endobronchial intima infection without casts (Figure 4). After six days, he was discharged with a little cough and no neurological sequelae; continued on oral azithromycin and nebulized budesonide treatment for two weeks. The timeline of this case was shown in Figure 5. During the next four follow-up visits, the patient was asymptomatic and without recurrent casts. Further, his chest CT scan showed increased lung markings (Figure 3B).

## Discussion

PB is also termed fibrinous bronchitis or cast bronchitis, and its presentation of PB is hallmarked by partial or complete airway blockage caused by casts, which induces the sudden onset of cough, wheezing, and progressive dyspnea, together with refractory hypoxemia. With this condition, pulmonary auscultation reveals wheezing and diminished breath sounds, with or without rales, as typical signs. Moreover, radiological examination reveals varying results, with consolidation and atelectasis being common, with the contralateral lung showing hyperinflation. Additionally, numerous cases present interstitial infiltrates, pleural effusion, pneumomediastinum, and pneumothorax [3,4]. All clinical presentations and radiological examinations are nonspecific, and PB diagnosis is confirmed via direct cast observation through bronchoscopy or expectoration.

Presently, there are several classification schemes for casts in PB; among them, the classification proposed by Seear et al. is the most widely used [1,2,11]. Specifically, Seear et al. proposed two types of bronchitis casts based on histology: type I and type II casts. Type I inflammatory casts are characterized by infiltrating inflammatory cells (mainly comprised of neutrophils, eosinophils), fibrin, mucin, and Charcot-Leiden crystals, and type II acellular casts are primarily comprised of mucin and a few fibrins [1]. These cast types are associated with different diseases. Specifically, type I casts are usually secondary to bronchial disease and present an acute course. Conversely, type II casts predominantly occur in children with CHD and are characterized by a chronic and recurrent course [11]. The case shown here was a type I PB cast, comprising inflammatory necrotic tissue, mucin, numerous neutrophils, and eosinophils.

Previous studies have reported bronchoscopy is the most direct and effective method for cast removal [4,5]. However, casts are sometimes sticky and brittle, and this impedes their extraction. Under these circumstances, adjuvant therapies, combined with bronchoscopy, are administered for cast removal [6,12]. Hypertonic saline, hyaluronic acid, and recombinant human deoxyribonucleases are common selections for adjuvant therapies in anecdotal cases [6,13]. However, there have been no reports of the use of α-chymotrypsin in PB treatment. Therefore, the use of α-chymotrypsin as an adjuvant treatment, of which this is the first reported case, maybe a novel schema for cast removal.

α-Chymotrypsin is a serine protease cleaved from chymotrypsinogen in the pancreas. Owing to its various biological activities, including anti-inflammatory, antioxidative, fibrinolytic, anti-edematous, and anti-infective effects, it has been widely used to accelerate the repair of traumatic injuries, burns, and to alleviate sciatica [13]. In addition, it has been used to treat chronic pulmonary disease and upper respiratory conditions [14]. Previous studies have demonstrated that it has a direct mucolytic effect on sputum. Specifically, it can liquefy the mucus and decrease the viscosity of sputum by destroying the peptide bridge of bi-polymers and hydrolyzing fibrin, mucin, and other sputum proteins into polypeptides or amino acids [14-16]; this favors the removal of purulent secretions and necrotic tissues. A decrease in the viscosity of sputum in patients with chronic bronchitis has also been demonstrated following the oral administration of α-chymotrypsin [16]. Additionally, α-chymotrypsin exerts an anti-inflammatory effect by increasing the expression levels of anti-inflammatory cytokines, such as IL-4 and IL-17, and decreasing the expression of IFN-γ in inflammatory lesions [17]. Moreover, chymotrypsin enhances the absorption and penetration of antibiotics and steroid hormones by increasing the tissue-fluid interchange [18]. To treat our patient, casts were successfully removed by instilling chymotrypsin on the casts. The success of this procedure may be attributed to the direct mucolytic effect of chymotrypsin in reducing the viscosity of casts and diluting the casts. Instilling chymotrypsin into the bronchus may reduce inflammation in the trachea and increase the permeability of other therapeutic agents, which favors follow-up treatments. Allergy is considered a major adverse reaction to chymotrypsin. However, our patient did not show any complications and improved his condition. Therefore, chymotrypsin can be considered safe and effective for other diseases. However, the limited case makes it insufficient. Further controlled clinical studies are needed to verify the efficacy of α-chymotrypsin in the treatment of PB.

## Conclusions

We present the case of a boy with sudden and progressive dyspnea who was found to have inflammatory casts in his bronchus. The initial extraction is partial and not remarkably successful. After instilled α- chymotrypsin into the bronchus, casts are extracted completely and successfully. Then, his condition improved and he got discharged. He shows asymptomatic and without recurrent casts on the follow-up visits. Clinicians should be highly alert to PB under the following circumstances. (a) Severe respiratory tract obstruction, ventilation dysfunction, and refractory hypoxemia occurred in a short time without a foreign body inhaled. (b) Pulmonary auscultation presents reduced respiratory sound of unilateral or bilateral lungs. (c) Chest radiologic examination shows atelectasis, compensatory emphysema, or pneumothorax. (d) There is no improvement by administering routine treatment, such as mechanical ventilation support, enhanced nursing sputum aspiration, and bronchodilators, while acute respiratory distress syndrome and acute lung injury cannot be explained. (e) Patients had expectorated jelly-like bronchial tree before. In such cases, aggressive treatment, including bronchoscopy and adjuvant therapies, such as α-chymotrypsin, is the key to saving lives and reducing sequelae.
